# Chronic Low Back Pain Masking Medullary Nephrocalcinosis Diagnosed by Point-of-Care Ultrasound

**DOI:** 10.7759/cureus.110368

**Published:** 2026-06-06

**Authors:** Jideofor K Ndulue, Chiagoziem J Ndulue, Chidozie N Ndulue

**Affiliations:** 1 Family Medicine, Southwest Illinois Family Medicine Residency, Saint Louis University School of Medicine, O'Fallon, USA; 2 Emergency Medicine, Hospital Sisters Health System St. Joseph's Hospital, Highland, USA; 3 Family Medicine, Treasure MD, O'Fallon, USA; 4 Nephrology, Nnamdi Azikiwe University Teaching Hospital, Nnewi, NGA

**Keywords:** low back pain, medullary sponge kidney, nephrocalcinosis, point-of-care ultrasound, renal calcifications

## Abstract

Low back pain (LBP) is a major cause of disability worldwide and is usually attributed to musculoskeletal etiologies. However, non-musculoskeletal causes should be considered in patients with persistent or atypical symptoms. Renal causes, such as renal calcific disorders, are less frequently considered, especially in the absence of urinary symptoms. Medullary nephrocalcinosis is a rare renal calcific disorder characterized by calcium deposition in the renal medullary parenchyma and tubules. We report the case of a 46-year-old woman with a three-year history of chronic LBP without urinary tract symptoms, managed conservatively as musculoskeletal pain, without improvement. Bedside point-of-care ultrasound (POCUS) revealed bilateral echogenic medullary pyramids suggestive of medullary nephrocalcinosis. Confirmatory imaging with computed tomography showed calcification of the medullary pyramids and multiple bilateral renal medullary cysts, consistent with medullary nephrocalcinosis and medullary sponge kidney. This case highlights an atypical presentation of medullary nephrocalcinosis and underscores the diagnostic utility of POCUS as a valuable, low-cost, noninvasive tool for assessing renal causes of LBP.

## Introduction

Low back pain (LBP) is a leading cause of disability globally, affecting 619 million people in 2020, and projected to rise to 843 million cases by 2050 [1]. It is among the most common presenting complaints in primary care and the emergency room [2,3]. Chronic LBP, defined as lumbar pain persisting for more than 12 weeks, affects 13% of adults in the United States, with 30% of those individuals experiencing significant disability and low quality of life [4].

Musculoskeletal factors, such as muscle sprain or strain, herniated disc, spinal stenosis, osteoarthritis, and vertebral compression fracture, are common causes of chronic LBP. However, non-musculoskeletal etiologies, including renal pathology, should be considered when symptoms are persistent or atypical. 

Medullary nephrocalcinosis refers to diffuse calcium deposition within the renal medulla. It is often asymptomatic or presents with nonspecific symptoms, leading to delayed diagnosis. Diagnosis is often an incidental finding on imaging, with computed tomography (CT) considered the gold standard. Point-of-care ultrasound (POCUS) has emerged as a rapid, non-invasive tool for early detection of renal pathology.

We present a case of medullary nephrocalcinosis identified by POCUS in a patient initially evaluated for chronic LBP.

## Case presentation

A 46-year-old woman presented to the emergency room with a three-year history of dull, bilateral, non-radiating LBP that had recently worsened. The pain was most intense in the lower right back, with no clear exacerbating or relieving factors. Initial treatment with physical therapy and conservative measures for presumed musculoskeletal pain resulted in minimal improvement. She reported occasional nausea but no history of fever or urinary tract symptoms.

Vital signs were within normal limits. Physical exam revealed mild right-sided paraspinal tenderness without costovertebral angle tenderness or neurologic deficits. Pertinent laboratory results include normal serum calcium, parathyroid hormone, and bicarbonate. Urinalysis showed no evidence of infection but was positive for calcium oxalate crystals.

**Figure 1:**
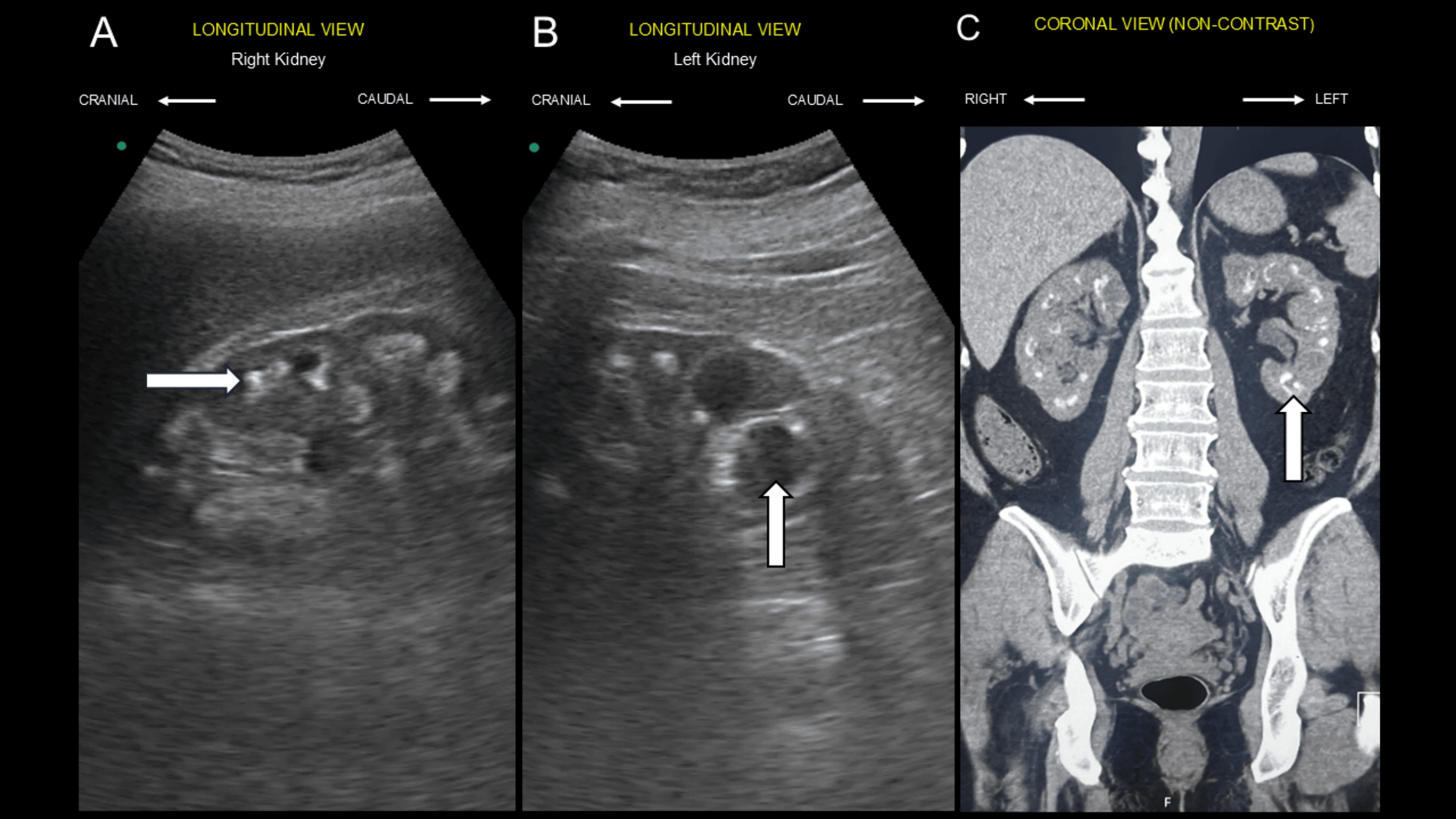
Renal imaging findings on point-of-care ultrasound and computed tomography (A) Point-of-care ultrasound of the right kidney demonstrating multiple medullary cysts, echogenic pyramids, and hyperechoic foci with posterior acoustic shadowing (arrow indicates a medullary cyst containing hyperechoic foci consistent with calculi). (B) Point-of-care ultrasound of the left kidney showing larger medullary cysts and calcifications (arrow indicates a medullary cyst containing hyperechoic foci consistent with calculi). (C) Non-contrast computed tomography of the abdomen demonstrating bilateral curvilinear calcifications of the medullary pyramids and medullary cysts consistent with nephrocalcinosis (arrow indicates medullary calcification/calculus).

A bedside renal ultrasound was performed using the Sonostar UProbe C5PL wireless ultrasound probe (SonoStar Technologies Co., Ltd., Guangzhou, Guangdong, China) with a 5.0 MHz curved array transducer. POCUS demonstrated multiple renal medullary cysts, echogenic medullary pyramids, and scattered hyperechogenic foci with posterior acoustic shadowing in both kidneys (Figures 1A-1B). Some of the hyperechogenic foci appeared embedded within the cysts.

Non-contrast CT of the abdomen revealed bilateral curvilinear calcification within the medullary pyramids along with multiple renal medullary cysts, consistent with medullary nephrocalcinosis in the setting of medullary sponge kidney (Figure 1C).

The patient received a liter of normal saline and intravenous ketorolac for pain control. She was referred to nephrology for further management.

## Discussion

This case demonstrates an uncommon presentation of medullary nephrocalcinosis, manifesting as chronic LBP over a prolonged period. The absence of urinary symptoms likely contributed to an initial diagnostic delay, highlighting the importance of maintaining a broad differential in persistent or atypical cases.

LBP may be secondary to renal calcific disorders, namely nephrolithiasis and nephrocalcinosis. Nephrocalcinosis refers to the deposition of calculi in the kidney parenchyma and tubules, whereas nephrolithiasis is the presence of calculi in the renal collecting system. Nephrocalcinosis usually involves the renal medulla, less often, the cortex [5]. Primary hyperparathyroidism, medullary sponge kidney, and distal renal tubular acidosis are the most common conditions associated with nephrocalcinosis [6]. Nephrocalcinosis is usually asymptomatic; it progresses slowly and is often discovered incidentally on abdominal or chest imaging, such as plain radiography, ultrasonography, or CT. It may also present as renal colic when associated with nephrolithiasis, hematuria, acute kidney injury, and, as in our case report, chronic back pain.

Imaging is essential for diagnosis. A plain abdominal X-ray and CT can show scattered renal calcifications. The sensitivity of abdominal X-rays is limited when calcium deposits are small [7]. On ultrasonography, nephrocalcinosis may appear as bright hyperechoic areas in the renal medulla and, less often, the cortex, indicating calcium deposits in the renal parenchyma [8]. A study found the highest sensitivity when combining ultrasound and CT for diagnosing nephrocalcinosis [9], whereas some studies have shown ultrasound to have higher sensitivity than CT, followed by X-ray [8,10]. A 2018 US nationwide study showed that a POCUS-first approach to evaluating renal calcific disorders could save $16.5 million by avoiding 159,000 non-contrast CT scans [11]. POCUS also led to a significant reduction in emergency room length of stay [11]. 

Although this report primarily focuses on diagnostic considerations, it is important to note that the evaluation of back pain in patients with medullary nephrocalcinosis should emphasize distinguishing renal causes, such as nephrolithiasis or urinary tract infection, from more common musculoskeletal etiologies, with management guided accordingly.

In this case, POCUS performed in the emergency setting was pivotal in identifying renal pathology and prompting further diagnostic evaluation. The findings correlated closely with CT imaging, reinforcing the utility of POCUS as an effective bedside diagnostic tool.

This case underscores the importance of considering non-musculoskeletal causes of chronic LBP, particularly when symptoms persist despite appropriate therapy.

## Conclusions

Medullary nephrocalcinosis can present atypically as chronic LBP in the absence of urinary symptoms. POCUS is a valuable diagnostic adjunct that can facilitate early identification of renal pathology. POCUS may be used as an initial imaging technique due to its portability, lack of radiation exposure, cost-effectiveness, and high sensitivity. Clinicians should maintain a broad differential diagnosis when evaluating persistent or refractory LBP.
